# A decade of whole-exome sequencing in Brazilian Neurology: from past insights to future perspectives

**DOI:** 10.1055/s-0045-1807715

**Published:** 2025-05-13

**Authors:** Caio Robledo D'Angioli Costa Quaio, Thiago Yoshinaga Tonholo Silva, Orlando G. Barsottini, Sarah Teixeira Camargos, Marcondes C. França, Jonas A. Saute, Wilson Marques, Fernando Kok, José Luiz Pedroso

**Affiliations:** 1 Hospital Israelita Albert Einstein, São Paulo SP, Brazil.; 2 Universidade de São Paulo, Faculdade de Medicina, Hospital das Clínicas, Instituto da Criança, São Paulo SP, Brazil.; 3 Universidade Federal de São Paulo, Escola Paulista de Medicina, Departamento de Neurologia, São Paulo SP, Brazil.; 4 Universidade Federal de Minas Gerais, Belo Horizonte MG, Brazil.; 5 Universidade Estadual de Campinas, Faculdade de Ciências Médicas, Departamento de Neurologia, Campinas SP, Brazil.; 6 Universidade Federal do Rio Grande do Sul, Faculdade de Medicina, Departamento de Medicina Interna, Porto Alegre RS, Brazil.; 7 Hospital de Clínicas de Porto Alegre, Serviço de Genética Médica, Porto Alegre RS, Brazil.; 8 Universidade de São Paulo, Faculdade de Medicina de Ribeirão Preto, Departamento de Neurociências e Ciências do Comportamento, Ribeirão Preto SP, Brazil.; 9 Mendelics Análise Genômica, São Paulo SP, Brazil.; 10 Universidade de São Paulo, Faculdade de Medicina, Departamento de Neurologia, São Paulo SP, Brazil.

**Keywords:** Exome Sequencing, Neurology, Molecular Diagnostic Techniques, Brazil

## Abstract

Over the last decade, whole-exome sequencing (WES) has become a standard diagnostic tool, significantly transforming the landscape of clinical genetics and playing a pivotal role in the diagnosis of neurogenetic diseases. This revolutionary shift has left a lasting impact on the field of neurology in Brazil. The current review article examines key developments and milestones achieved in Brazil through the application of WES in neurology and discusses forthcoming challenges and essential steps to advance molecular diagnosis. Several studies report the use of WES to diagnose genetic disorders with neurological manifestations in Brazil, underscoring the growing importance of molecular diagnosis in neurogenetics. These studies often provide detailed phenotypic analyses and clinical descriptions, offering valuable insights into the genetic underpinnings of several neurological conditions. Many reports highlight the use of WES in the investigation of complex neurological conditions in Brazil, such as neurodevelopmental disorders, hereditary spastic paraplegia, movement disorders, and ataxia. The discovery of new genes implicated in monogenic diseases with neurological manifestations through WES was a significant breakthrough. Despite these advances, the availability of large cohort studies on rare diseases in Brazil remains limited, hindering the ability to generalize findings and explore the full spectrum of genetic diversity. However, a few larger cohort studies have substantially contributed to our understanding of rare diseases and specific neurological disorders.

While WES has limitations and may eventually be supplanted by more advanced diagnostic tools, it left a permanent mark on the neurology field in Brazil. The field of neurogenetics is set to become increasingly important in the future.

## INTRODUCTION

Over the last decade, the incorporation of whole-exome sequencing (WES), also simply referred to as *exome sequencing* (ES), as a standard diagnostic tool has profoundly transformed the landscape of clinical genetics, playing a pivotal role in the diagnosis of neurogenetic diseases. 1 2 3 4 5 6 7 8 9 10 This revolutionary shift in molecular diagnosis has left a permanent mark on the neurology field in Brazil.

Before the advent of massive parallel sequencing (MPS) technologies, achieving an early genetic diagnosis for patients with rare diseases was often a costly endeavor, frequently involving unnecessary procedures, tests, and medical consultations. 1 This “diagnostic odyssey” could be attributed to several factors. Firstly, rare diseases form a vast and heterogeneous group of more than 6 thousand distinct conditions, most of which have a genetic basis. 2 The approach of clinical diagnosis followed by gene-specific molecular analysis proved challenging, as many of these conditions exhibit common, vague, or overlapping symptoms.

The landscape of diagnostics was revolutionized by the introduction of large-scale genomic studies. Based on MPS technology, WES emerged as an efficient method for molecular diagnosis in patients with rare diseases. By enabling the simultaneous sequencing of most of the coding regions of the human genome, WES facilitates the comprehensive examination of most genes known to be associated with genetic disorders. 3 This method significantly shortens the journey to identify the molecular cause of a rare disease, providing an etiological diagnosis that can lead to transformative health outcomes. 4 This includes the adaptation of pharmacotherapy, target therapies, appropriate specialist referrals, the elimination of unnecessary interventions, the discontinuation of ineffective treatments, and the initiation of palliative care, marking a pivotal shift in the management of patients with rare diseases. 5 6 7

The present article provides a review of key developments and milestones achieved in Brazil through the application of ES in neurology and discusses some forthcoming challenges and critical steps that are essential to advance molecular diagnosis in this field. The detailed methodology of the current study, including the process of article selection and review, can be found in the Supplementary Material (avalailable at: https://www.arquivosdeneuropsiquiatria.org/wp-content/uploads/2025/01/ANP-2024.0212-Supplementary-Material.docx )

## EXOME SEQUENCING IN GENETIC DISEASES

Also called *next-generation sequencing* (NGS) or *high-throughput sequencing* , MPS represents a revolutionary advancement in the field of genomics. It has transformed the way researchers and clinicians examine an individual's genomic information by enabling the rapid and cost-effective analysis of hundreds or even thousands of different genomic regions in one single test. 8

Although MPS presents several different applications, it follows certain standard steps. Firstly, DNA samples are extracted from the biological material of interest, fragmented into smaller pieces, and ligated to adapters (specific DNA fragments) that are important to sequence and index the samples. 9

**Figure 1:**
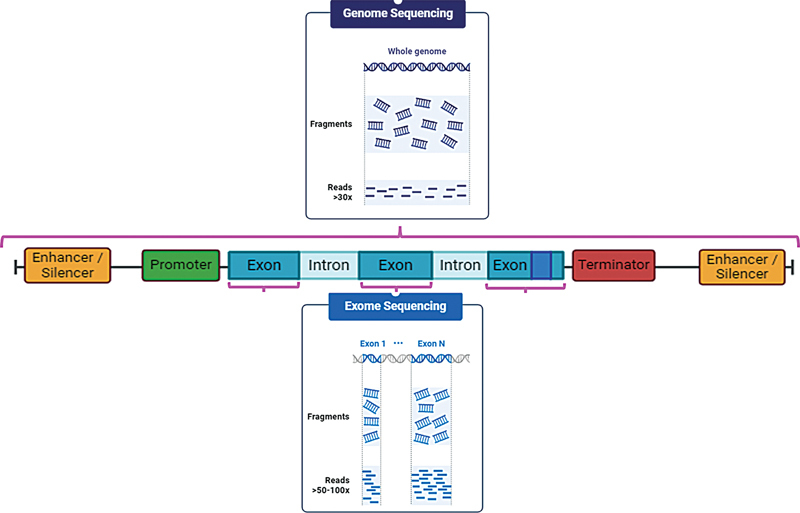
Schematic figure of the gene, including the non-coding elements (enhancer/silencer and promoter), introns, and exons. Exons correspond to the coding part of the gene that is translated to a protein; they correspond to approximately 2% of the genome, but cause most of the known genetic disorders. Whole-exome sequencing (WES) can only identify variants in the exons. Whole-genome sequencing (WGS) can identify variants in any part of the genome, including the non-coding DNA.

Then there is a capture step, in which specific DNA fragments of interest (hundreds or even thousands, depending on the purpose of the study) are isolated and enriched before sequencing. This step is essential for targeted sequencing approaches, including targeted-gene panels and WES. 10 For targeted-gene panels, the capture of DNA sequences is restricted to a specific set of genes of interest; in contrast, capture for WES targets most of the human exome ( Figure 1 ), an approach that provides a broader genomic analysis and is particularly useful to explore genetic disorders with complex genetic bases. 9

These steps, which are key stages in library preparation, aim to convert a genomic DNA sample (or cDNA sample) into a library of fragments that can subsequently be sequenced. Variability in library preparation among different laboratories can directly affect the consistency, reliability, and comparability of the results. The Brazilian Society of Medical Genetics and Genomics (Sociedade Brasileira de Genética Médica e Genômica, SBGM, in Portuguese) is actively promoting a working group dedicated to advancing efforts toward more standardized library preparation. This initiative aims to enhance the reproducibility of findings across laboratories and improve diagnostic accuracy.

Following the aforementioned steps, certain protocols may include an amplification step using polymerase chain reaction (PCR) to increase the amount of DNA available for sequencing. The prepared samples are then ready for sequencing and may be loaded into a sequencer (such as Illumina [Illumina, Inc., San Diego, CA, United States], Ion Torrent [Thermo Fisher Scientific, Waltham, MA, United States] or other MPS platforms) which reads, depending on the platform, the fluorescent, electrical or other signals generated as nucleotides are incorporated. 1 These signals are processed by bioinformatics protocols to determine the sequence of nucleotides at each position, a step known as *base calling* .

The sequence data (stored in FASTQ files) may then be further processed: every short segment of sequence (also called *read* , which is a sequence generally composed of 100 to 150 nucleotides) is aligned to the human reference genome. Genetic variations may now be detected, a step known as *variant calling* . This step presents potential challenges, such as the considerable computational resources required to deal with large amounts of data and sophisticated algorithms to accurately align sequences and call variants among the noise.

Other information from several databases (such as frequency in controls, functional impact, and in-silico predictions) may be annotated to provide relevant information for the last step of the process: clinical analysis, variant classification, and reporting.

The clinical analysis of MPS data requires clinical expertise and a complex set of bioinformatics methods designed to interpret the massive volumes of DNA sequence information generated by these new sequencing technologies, some of them previously explained herein. One of the main complexities of the analysis refers to variant prioritization, which corresponds to distinguishing clinically-relevant variants from benign polymorphisms. 10 11 12 Therefore, deep understanding of genetics and a bit of medical “art” are often required to unravel a molecular diagnosis.

Once a variant is identified as clinically relevant, a systematic approach is undertaken to determine its significance in relation to a disease. This process involves gathering relevant evidence, such as clinical information available in previous reports from the literature and scientific databases (such as ClinVar), frequencies from population control databases (such as gnomAD and the Brazilian ABraOM), in-silico prediction tools, segregation analysis, and studies on functional effects, among others. These pieces of evidence are then used to classify the variant into one of the following categories: pathogenic, likely pathogenic, variants of uncertain significance (VUS), likely benign, and benign. This classification step is crucial to standardize the assessment of the impact of specific genetic variants on health outcomes across different genetic services.

The American College of Medical Genetics and Genomics (ACMG), together with the Association for Molecular Pathology (AMP), have significantly reorganized and standardized the process of variant classification by providing a comprehensive framework to evaluate the pathogenicity of genetic variants. 12 This standardization has improved accuracy in interpreting genetic findings and also enhanced communication between geneticists, clinicians, and patients by providing a common language and a more uniform process. Several modifications to the original framework have been proposed by various genetics societies and initiatives, such as ClinGen, in order to keep pace with the rapidly evolving field of genetics, although they have also increased the complexity of the process.

Following clinical analysis and variant classification, a clinical report of the relevant genetic variants will be generated. This is a detailed document that provides information about the identification, analysis, classification, and interpretation of the genetic variants detected through genetic testing, within the context of a patient's clinical presentation and family history. This document is intended to improve clinical decision-making by healthcare providers.

## APPLICATION OF MPS IN NEUROLOGY AND ITS CLINICAL IMPACT

The close relationship between genetics and neurological disorders has been recognized since the origin of neurology. However, until MPS was thoroughly widespread, only a few disorders had received the molecular diagnosis. Previously, the investigation of monogenic conditions caused by sequence variants relied exclusively on single-gene sequencing using the Sanger technique, which required prior clinical suspicion of a specific gene or a very restricted subset of genes. The WES techniques have been used in the investigation of neurological disorders for over a decade, and their clinical usefulness was rapidly proven. Whole-exome sequencing has enabled the identification of an exponential growing number of genes causative of neurological diseases, and it has widened the genotype-phenotype relationship of several others, with a more cost-effective yield than that of previous technologies. 8 13

Besides sequence variants (including single-nucleotide variants [SNVs] and indels [insertions and deletions]), MPS has also been proven to be highly effective in the study of total aneuploidies and partial aneuploidies, including those detectable at microscopic and submicroscopic resolutions. This is achieved through bioinformatics protocols that compare the coverage patterns of chromosome segments across different samples. 14 Therefore, WES has become a highly-powerful tool for the diagnostic investigation of monogenic diseases, effectively identifying both deleterious sequence variants and copy number variations (CNVs).

Whole-exome sequencing has demonstrated a higher diagnostic yield compared to that of targeted-gene panels. The latter are limited in scope, focusing only on select sets of genes implicated in specific diseases, which restricts their diagnostic capability. Consequently, targeted-gene panels may miss up to 64% of molecular diagnoses for rare diseases that could be identified through WES. 15 16 However, targeted-gene panels may offer advantages over WES in certain aspects, such as reduced cost, shorter turnaround time, absence of secondary findings, and higher coverage of targeted genes.

Due to its remarkable diagnostic power, WES has been recommended as a first-line diagnostic test, particularly in the pediatric population. 17 18 It has been proven to be highly clinically useful, significantly impacting patient management and outcomes. A meta-analysis 19 of the diagnostic and clinical usefulness of WES and genome sequencing demonstrated that WES influenced clinical management in 48% of the patients diagnosed through this technique. The impacts included enhanced surveillance, referrals to specialists, modifications to diet and lifestyle, hospitalizations, and determining the appropriateness of various investigations, procedures, surgeries, and medications. Beyond these clinical benefits, the application of WES also has notable economic implications for the healthcare system. By accurately diagnosing conditions, WES helps avoid unnecessary procedures and hospitalizations, thereby reducing overall healthcare costs. 19 20 21 Recent advances in sequencing techniques have also enabled the current development of gene-targeted therapy with drugs designed to act in a specific genotype, which include gene therapy, gene-editing techniques, and oligonucleotide therapies. Currently, these gene-targeted therapies are costly and limited to a few disorders, but great effort is being invested, with the expectation of a tailored treatment for many monogenic diseases. 22

The diagnostic approach of monogenic diseases, particularly in neurology, has been revolutionized by MPS. However, many challenges must be faced to improve the quality of healthcare. The first challenge is the improvement of variant classification to minimize the discrepancies among different investigators, and to better understand the disease mechanism, especially in non-coding regions, enabling a more accurate interpretation of the impact of the result in clinical practice. Another major challenge that must be addressed is the ethical issues derived from the genetic data, including presymptomatic diagnosis in a healthy individual, and its impact in psychological, familial, social, and labor affairs; the edge between prevention and eugenics, concerning reproductive medicine; and genetic testing of children and non-complying individuals. Lastly, the high cost and the consequent limited access to MPS is expected to be overcome with the improvement of the technology. 23

Over the past decade, MPS has significantly accelerated the pace of novel gene discoveries, with the rate of discovery nearly tripling compared to conventional methods. 21 In recent years, more than 250 new disease-associated genes have been identified annually. 1 Consequently, over 4,600 genes are currently linked to Mendelian disorders, and the discovery of new genes continues at a steady pace.

## BRAZILIAN PERSPECTIVE AND INITIATIVES

There are several reports of the use of WES for the diagnosis of genetic disorders with neurological manifestations in Brazil, highlighting the growing importance of molecular diagnosis in the field of neurogenetics. 24 25 26 27 28 29 30 31 32 33 34 35 36 37 38 39 40 41 42 43 44 45 46 47 48 49 50 51 52 53 54 55 56 57 58 59 60 61 62 63 64 65 66 67 68 69 70 71 72 73 74 75 Some of the main Brazilian scientific contributions are summarized in Table 1 . These reports often emphasize detailed phenotypic analyses and clinical descriptions, providing valuable insights into the genetic underpinnings of a wide array of neurological conditions.

**Table 1:** Summary of the main Brazilian scientific contributions that have employed exome sequencing in the field of neurology

Author	Year	Study population	Type of article	Number of individuals or families	Rate of diagnostic yield (if applicable)
**Cohorts exploring the diagnostic yield fo exome sequencing**					
Souza et al. 57	2017	Hereditary spastic paraplegia	Case series	21	62%
Carneiro et al. 58	2018	Syndromic neurodevelopmental disorders	Case series	8	62%
Homma et al. 59	2019	Rare diseases/syndromic	Case series	44	34%
Montenegro et al. 60	2020	Syndromic neurodevelopmental disorders	Case series	30	23%
Quaio et al. 61	2020	Rare diseases/syndromic	Case series	500	32%
Giordani et al. 63	2021	Hereditary spastic paraplegia	Case series	106	64%
Gonçalves et al. 64	2021	Neuropathies and neuronopathies	Case series	107	70%
Carvalho et al. 65	2022	Syndromic neurodevelopmental disorders	Case series	20	47%
Graça et al. 66	2022	Ataxia	Case series	76	36%
Leite et al. 67	2022	Neurodevelopmental disorders	Case series	369	42%
Tacla et al. 68	2024	Rare diseases/syndromic	Case series	17	35%
Chaves et al. 69	2023	Syndromic neurodevelopmental disorders	Case series	11	73%
Fussiger et al. 70	2023	Hereditary spastic paraplegia	Case series	95	60%
Novis et al. 71	2024	Ataxia	Case series	76	16%
Figueiredo et al. 72	2024	Neuropathies and neuronopathies	Case series	53	68%
Silveira et al. 73	2024	Syndromic neurodevelopmental disorders	Case series	43	7%
Tolezano et al. 74	2024	Syndromic neurodevelopmental disorders	Case series	45	45%
**Experimental studies using exome sequencing**					
Vieira et al. 44	2014	Limb-girdle muscular dystrophy	Experimental	28	−
Figueiredo et al. 47	2015	Syndromic neurodevelopmental disorders	Experimental	9	−
Melo et al. 45	2015	Hereditary spastic paraplegia	Experimental	2	−
Cappi et al. 50	2016	Neurodevelopmental disorders	Experimental	20	−
Figueiredo et al. 48	2016	Syndromic neurodevelopmental disorders	Experimental	7	−
Lima et al. 51	2016	Neurodevelopmental disorders	Experimental	30	−
Cappi et al. 52	2020	Neurodevelopmental disorders	Experimental	222	−
Garcia-Rosa et al. 49	2019	Multiple sclerosis	Experimental	1	−
Souza et al. 55	2020	Myopathies	Experimental	12	−
Zanardo et al. 53	2020	Syndromic neurodevelopmental disorders	Experimental	38	−
Quaio et al. 62	2022	Rare diseases/syndromic	Experimental	500	−
Raposo et al. 54	2022	Ataxia	Experimental	4	−
Rebelo et al. 46	2023	Neuropathies and neuronopathies	Experimental	9	−
**Other cohorts**					
Abrahao et al. 25	2016	Dementia	Case series	3	−
Moreno et al. 29	2016	Myopathies	Case series	7	−
Pinto et al. 34	2019	Neuropathies and neuronopathies	Case series	7	−
Souza et al. 38	2019	Inherited metabolic diseases	Case series	5	−
Garcia et al. 24	2020	Hemimegalencephaly	Case series	5	−
Monteiro et al. 42	2020	Rare diseases/syndromic	Case series	3	−
Carvalho LML et al. 65	2022	Syndromic neurodevelopmental disorders	Case series	2	−
Freua et al. 35	2022	Inherited metabolic diseases	Case series	7	−
Prota et al. 26	2022	Dementia	Case series	2	−
Brito et al. 31	2023	Neuropathies and neuronopathies	Case series	4	−
Maciel et al. 32	2023	Neuropathies and neuronopathies	Case series	2	−
Barcelos et al. 39	2023	Interferonopathy	Case series	6	−

A particularly illustrative group benefiting from WES studies encompasses the neurodevelopmental disorders, including autism spectrum disorders and intellectual disability, in which extensive research and reports have helped to clarify phenotypic spectrums of several monogenic forms. Additionally, clinical reports and family studies 24 25 26 27 28 29 30 31 32 33 34 35 36 37 38 39 40 41 42 43 have increasingly reported the use of WES for the investigation of a variety of complex neurological conditions in Brazil, including hereditary spastic paraplegia, movement disorders, epilepsy, ataxia, hemimegalencephaly, inherited dementia, myopathies, neuropathies and neuronopathies, inherited metabolic diseases, Aicardi-Goutières syndrome, leukoencephalopathies, primary brain calcification, and complex neurological phenotypes.

Through WES, Brazilian neurologists and geneticists have been able to identify novel variants and establish genetic diagnoses for disorders that were previously unexplained, opening new avenues to understand disease mechanisms and to develop patient management protocols, and potential therapeutic strategies. This molecular approach also extends beyond the immediate clinical implications, offering a unique window into the mosaic that constitutes the Brazilian genetic backbone. Researchers have begun to map the landscape of Brazilian genetic variants, uncovering the genetic diversity that defines our population. This diversity derives from a complex history of miscegenation of indigenous, European, and African populations, and, more recently, global migration, resulting in a truly admixed population, each contributing to a unique genetic signature. Identifying and cataloging these variants not only enables a better understanding of genetic disorders but also helps in the recognition of population-specific variants. 56 Mapping the unique Brazilian genetic variants may help overcome a significant difficulty in the genetic testing of non-European ancestry groups: current genomics databases have limited representation for these populations. This limitation is particularly pronounced in the populations of lower- and middle-income countries, where the lack of comprehensive genetic data exacerbates the challenge of accurately interpreting test results and may lead to a much higher frequency of variants of uncertain significance.

Despite these advances, a significant gap remains in the availability of large cohort studies of rare diseases within the Brazilian context. Most studies tend to be limited in scale, focusing on individual cases or small groups of patients. This limitation hampers the ability to generalize findings across the broader population and to explore the full spectrum of genetic diversity and its impact on neurological disease presentation and progression in Brazil. This challenging landscape may be mitigated through the implementation of various strategies, such as establishing national and international collaborative research networks, investing in biobanks, and using digital platforms to integrate existing national databases. With adequate funding, public engagement initiatives, and policy support, these approaches can lay the foundation for large-scale rare-disease studies in Brazil.

However, it is noteworthy that there are exceptions regarding this trend, with a few larger cohort studies making substantial contributions to our understanding of rare diseases and specific neurological disorder groups. We will explore some of these studies as follows.

### Discovery of novel Mendelian diseases, new mechanisms of diseases, and other experimental studies

A combined approach using WES and other MPS techniques has led to the groundbreaking discovery of new genes implicated in limb-girdle muscular dystrophy, 44 hereditary spastic paraplegia, 45 and distal hereditary motor neuropathy with upper neuron involvement. 46 Whole-exome sequencing was also employed in the identification of genes associated with new forms of monogenic neurodevelopmental disorders. 47 48 This technique has also been employed in experimental studies exploring the genomic basis and pathophysiology of multiple sclerosis 49 and mental diseases, such as attention-deficit hyperactivity disorder 50 51 and obsessive-compulsive disorder. 52 Other experimental studies using WES have analyzed the detection power of CNVs. 53 Additionally, WES has been employed to investigate the modulation of phenotypes in conditions like Machado-Joseph disease 54 and myopathies. 55

The variant interpretation field has also benefited from notable Brazilian contributions. Over the past decade, the classification of variants has undergone considerable progress, notably through the adoption of standardized guidelines. 12 The rapid evolution of genetics research and the growing complexity in interpreting genomic data require periodic updates to these guidelines. Additionally, the genetic diversity of the Brazilian population, with its complex admixture, introduces challenges in terms of interpreting variants, especially those of uncertain significance. A proposal for a Brazilian standard for the classification of constitutional sequence variants has been put forward, 75 tailored to the unique genetic characteristics of the Brazilian population. This initiative also moves towards incorporating more quantitative criteria and the use of Bayesian statistical reasoning in the interpretation process.

### Neurodevelopmental disorders

In a study 58 on eight probands with sporadic syndromic intellectual disability and their parents, WES revealed likely pathogenic variants in eight candidate genes. These included biallelic variants in three autosomal genes ( *ADAMTSL2* , *NALCN* , *VPS13B* ), one in an X-linked gene ( *MID1* ), and de novo heterozygous variants in four autosomal genes ( *RYR2* , *GABBR2* , *CDK13* , *DDX3X* ). In 62% of the cases, the variants explained the clinical findings. The authors 58 concluded that, while causative recessive variants could be identified without parental sequencing, the trio-based approach was crucial to identify de novo dominant variants, highlighting the importance of including parental samples to increase the diagnostic yield.

In a separate study 60 on 30 patients with autism spectrum disorder and their parents, researchers assessed the role of de novo variants using data from a meta-analysis with more than 20 thousand neurodevelopmental disorder patients. The researchers 60 identified 3 pathogenic CNVs and 7 de novo sequence variants in known genes, alongside 12 de novo variants in previously-unidentified autism genes. The study proposed *PRPF8* and *RBM14* as novel candidates for neurodevelopmental disorders, achieved a diagnostic yield of 23%, and highlighted the value of the unique Brazilian cohort in identifying and validating potential new genetic loci for autism.

In a study 65 on syndromic obesity, which includes obesity alongside intellectual disability and developmental delay, WES trios on 20 patients revealed pathogenic or likely pathogenic variants in 6 cases, involving genes such as *MED13L* , *AHDC1* , *EHMT1* , *MYT1L* , *GRIA3* , and *SETD1A* , while 2 patients had inherited variants of uncertain significance. Additionally, pathogenic or likely-pathogenic CNVs were detected in 3 out of 15 patients, involving *SATG2* , *KIAA0442* , and *MEIS2* . Notably, all nine pathogenic variants were in genes known to cause syndromic neurodevelopmental disorders, but only *EHMT1* and *MYT1L* have established links to obesity. This study 65 achieved a diagnostic yield of approximately 47% and suggested that several known neurodevelopmental disorder genes may also predispose individuals to obesity.

In a cohort of 369 patients with intellectual disability, 67 combined MPS and chromosomal analyses determined the genetic etiology in 156 cases (42.3%). The Brazilian authors 67 underscored the effectiveness of integrating multiple diagnostic approaches to enhance the identification of genetic contributors to intellectual disabilities.

In an analysis of X-chromosome inactivation patterns in 194 women with intellectual disability, researchers 69 identified extreme or total skewing in 11 cases (8%). Extreme skewing of X-chromosome inactivation can be associated with neurodevelopmental disorders caused by pathogenic variants on the X chromosome. Subsequent WES revealed pathogenic variants in 8 (73%), including 4 in the X-linked genes ( *DDX3X* [2 cases], *WDR45* , and *PDHA1* ), and 4 in the autosomal genes ( *KCNB1* , *CTNNB1* , *YY1* , and *ANKRD11* ), all associated with dominant neurodevelopmental disorders. 69

In a study 73 on 43 individuals with clinical features consistent with 22q11.2 deletion syndrome, 3 (6.7%) were found to have heterozygous pathogenic variants in the *KMT2A* gene, including a novel variant and 2 previously-reported ones. Reverse phenotyping confirmed these patients had Wiedemann-Steiner syndrome, leading the authors 73 to propose that 22q11.2 deletion syndrome and Wiedemann-Steiner syndrome should be considered in each other's differential diagnoses due to overlapping clinical features.

Finally, a recent study 74 on syndromic microcephaly and neurodevelopmental disorders in a Brazilian cohort of 45 individuals negative for pathogenic CNVs identified pathogenic or likely-pathogenic variants in 19 families across 18 genes, achieving a diagnostic yield of 45%. Most participants presented with global developmental delays or intellectual disabilities (86%), facial dysmorphisms (80%), behavioral issues (> 50%), brain malformations (67%), musculoskeletal or cardiovascular defects (71% and 47%, respectively), and short stature (54%), reflecting the complexity and variability of these disorders.

### Hereditary spastic paraplegia

In a study 57 involving 21 patients from 17 Brazilian families with complex, heterogeneous hereditary spastic paraplegia (HSP) phenotypes, WES was used after previous negative results for SPG11/ *KIAA1840* and *SPG7* mutations, and it provided a definitive genetic diagnosis in 13 patients from 12 unrelated families, a diagnostic yield of 62%. The findings expanded the clinical spectrum of HSP, uncovered new pathophysiological mechanisms, and suggested potential therapeutic targets.

A large study 63 across five centers in Brazil examined 106 individuals from 83 families with suspected childhood-onset HSP and identified a molecular diagnosis in 68 individuals (diagnostic yield of 64%) from 50 families. Spastic paraplegia type 4 (SPG4) was the most common subtype, followed by type 3A (SPG3A). Both subtypes were marked by slow neurological progression. These findings were highlighted as key to understanding the molecular and clinical epidemiology of childhood-onset HSP, improving differential diagnosis, patient care, and paving the way for future collaborative trials.

In a study 70 on 95 Brazilian index cases of HSP, the researchers assessed the frequency of CNVs in the *SPAST* and *ATL1* genes using a combined approach of multiplex ligation-dependent probe amplification (MLPA) and MPS techniques. Diagnoses were established in 57 cases (60%), with SPG4 accounting for 26.3%. The most frequent autosomal recessive subtypes identified were type 7 (SPG7), followed by type 11 (SPG11), type 76 (SPG76), and cerebrotendinous xanthomatosis. Interestingly, no CNVs were found in the *SPAST* and *ATL1* genes. The authors 70 concluded that CNVs are rare among SPG4 and SPG3A families in Brazil, and they suggested that using specific algorithms to detect CNVs from MPS data is a cost-effective approach in low-risk populations, with MLPA reserved as a confirmatory test.

### Ataxia

A study 66 applied WES to a cohort of 76 adult patients with ataxia who had previously tested negative for trinucleotide repeat expansions. Whole-exome sequencing provided a diagnosis for 35.5% of the cases (25 patients), revealing a wide range of genetic causes. Notably, variants in two specific genes, *COQ8A* and *TTPA* , were discovered, and these forms of ataxia are prone to target therapies with coenzyme Q10 and vitamin E. These findings underscore the value of WES in diagnosing potentially-treatable ataxias.

Another study 71 combined WES with ExpansionHunter (free and open source), a bioinformatics tool designed to detect specific genetic expansions, in 76 diverse Brazilian families presenting ataxia. A definitive diagnosis was achieved in 16% of the cases, with probable diagnoses in an additional 16%. Notably, *RFC1* -related ataxia emerged as the most prevalent form within the cohort, closely followed by variants in *KIF1A* and *SYNE1* . An interesting finding was that early-onset cases of ataxia were more likely to receive a diagnosis; additionally, the study 71 also reported a 4% increase in diagnostic rates attributable to the use of ExpansionHunter.

### Neuropathies and neuronopathies

A study 64 on the genetic basis of familial amyotrophic lateral sclerosis (ALS) with 107 affected patients employed a 3-step diagnostic approach: triplet repeat primed PCR for *C9orf72* expansions; fragment digestion for the c.166 C > T *VAPB* variant; and WES.

A genetic cause was identified in 70% of the sample, with *VAPB* and *C9orf72* being the most frequent genes (in 30% and 22% of the subjects, respectively), followed by *SOD1* , *TARDBP* , *ANXA11* , and *FUS* . Five novel variants in known ALS genes were found. The authors 64 of the study concluded that *VAPB* and *C9orf72* are the genes most commonly related to familial ALS in Brazil.

In a study 72 on 53 pediatric patients with various forms of neuropathy, an impressive molecular diagnostic success rate, of 68% (36 patients), was achieved through the application of mixed MPS tests, including WES. Variants in *MFN2* and *GJB1* were the most common, together accounting for half of the confirmed diagnoses. The authors 72 highlighted the significant genetic heterogeneity observed, attributing it to the diverse ethnic background of the Brazilian population.

### Other rare diseases with neurological manifestations

A study 59 on the genetic causes of syndromic short stature in 44 children, many with neurodevelopmental disorders and other neurological manifestations, revealed that 15 patients (34%) had pathogenic or likely-pathogenic variants in genes associated with growth disturbances. The authors 59 concluded that the rarity and heterogeneity of syndromic short stature make clinical diagnosis challenging, but WES may effectively diagnose patients with these conditions.

One of the largest Brazilian studies 61 on rare diseases applied WES to a cohort of 500 patients, primarily with neurological manifestations, achieving a diagnostic yield of 31.6%. Notably, 38% of the identified variants were rare and previously unreported. The diagnostic rate varied with age, with higher yields in prenatal samples (67%) and infants (44%) compared to adults older than 50 years of age (13%). Despite these successes, 68.4% of the cohort remained undiagnosed, reflecting ongoing challenges in rare disease genomics. 61 Additionally, WES revealed secondary findings in 7.4% of patients, identifying actionable conditions such as hereditary cancer, arrhythmias, metabolic disorders, and cardiomyopathies. 62

A study 68 on 17 individuals with orofacial clefts and microphthalmia/anophthalmia/coloboma, many also with neurodevelopmental disorders, used chromosomal microarray and WES. While microarray did not detect any relevant CNVs, WES provided a conclusive diagnosis in 6 individuals (35.29%), identifying variants in the *CHD7* , *TFAP2A* , *POMT1* , *PTPN11* , and *TP63* genes, associated with the following syndromes: CHARGE, *CHD7* -spectrum, branchiooculofacial, *POMT1* -spectrum, LEOPARD, and ADULT. Variants of uncertain significance potentially associated with the phenotypes were found in six other individuals.

## CHALLENGES AND BARRIERS TO THE WIDESPREAD ADOPTION OF WES IN BRAZIL

There are several challenges to the widespread adoption of WES in Brazil. These include the need for complex, expensive equipment and trained personnel for data interpretation and clinical application. Specific obstacles include the geographical maldistribution of resources, lack of reimbursement in the public sector, limited awareness and education among key stakeholders, a shortage of specialists, and a paucity of local data for variant interpretation. 76

One significant challenge for the widespread adoption of WES is “acceptability”: patients or communities may decline testing due to fears about the potential results or because they perceive a lack of benefit and prefer to live with uncertainty. Additionally, there may be concerns about being exploited, particularly regarding how their genetic information might be used or shared. These apprehensions can stem from a mistrust of the government or healthcare system, cultural beliefs, or a lack of understanding about the potential advantages of genetic testing. Addressing these concerns through education, transparent communication, and community engagement is crucial to improve the acceptance and use of WES.

There are other important ethical considerations to address. Low literacy levels can impact the informed consent process, making it essential to ensure that participants fully understand the implications of genetic testing. This may require tailored communication strategies, such as the use of visual aids and simplified language. Furthermore, safeguarding data privacy and preventing genetic discrimination are critical, particularly in the context of socioeconomic inequality.

Thus, broad access to WES in Brazil remains limited, particularly for most the population that relies exclusively on the Unified Health System (Sistema Único de Saúde SUS, in Portuguese). While SUS theoretically provides universal health coverage, the availability of genetic testing within this system is still highly constrained. Access to these tests is predominantly available through private healthcare, research initiatives, or judicial intervention. Furthermore, there are significant gaps in policies for establishing a comprehensive diagnosis program using WES.

The SUS has recently made some policy advances, such as the Clinical Protocol and Therapeutic Guidelines (Protocolos Clínicos e Diretrizes Terapêuticas, PCDT, in Portuguese) for exome sequencing in cases of intellectual disability, which is currently the only indication for WES in the public sector. 77 Even with this policy and the broader framework provided by Ordinance no. 199 of the Brazilian Ministry of Health, which established the National Policy for Comprehensive Healthcare to Individuals with Rare Diseases (Política Nacional de Atenção Integral às Pessoas com Doenças Raras, PNAIPDR, in Portuguese), the incorporation of WES by most public reference centers for rare diseases under the SUS has not yet occurred. To date, WES is only offered in the clinical setting by a few institutions such as Universidade Federal do Rio de Janeiro (UFRJ), Fundação Oswaldo Cruz (Fiocruz), and, more recently, by Hospital de Clínicas de Porto Alegre (HCPA). At HCPA, the indication for WES is still limited to the investigation of intellectual disabilities, with sample collection having started in October 2023 and the first reports expected in January 2024 (courtesy of JAS, one of the authors of the present study).

Coverage of WES in the supplementary health sector, which includes private health insurance, is regulated by Appendix II of the Use Guidelines for the List of Procedures and Events in Health (Anexo II, DUT, Rol de Procedimentos e Eventos em Saúde, in Portuguese; RN 465/2021 and is limited to individuals with negative microarray investigation who present with at least two of the following: intellectual disability or neurodevelopmental delay; one major or three minor congenital anomalies; and short stature or failure to thrive. 78

The current article highlights the needy for the SUS and the supplementary health sector to incorporate genetic tests more broadly, given their significant benefits. Advocating for the inclusion of WES in the SUS could help pressure policymakers to expand access, ensuring that these essential diagnostic tools are available to all Brazilians, regardless of their income.

## THE NEXT FRONTIERS IN NEUROLOGY FOR MOLECULAR DIAGNOSIS

While a powerful tool in genetic diagnostics and research, WES presents certain limitations in the study of neurological disorders that are summarized in Table 2 . Firstly, once WES targets the coding regions of the genome, which comprise only about 1 to 2% of the entire genome, it is likely to miss alterations in non-coding regions that play crucial roles in gene regulation and expression. 79 Secondly, WES also has a limited ability to detect structural variations (SVs), such as smaller CNVs, inversions, insertions, or deletions, and it presents limitations in terms of identifying indels larger than 100 base pairs. 1 Thirdly, the method uses short-read sequencing, which can struggle with regions of the genome that are highly repetitive or rich in guanine-cytosine (GC) content, leading to gaps in sequencing coverage or misalignments. 80 The latter compromises the identification of pathogenic expansions, a molecular mechanism very important in neurogenetics, as it underlies the origin of several genetic diseases, including Huntington's disease, the highly prevalent Machado-Joseph disease, other spinocerebellar ataxias, and many more. Finally, the architecture of bioinformatics protocols is primarily designed to identify variants associated with monogenic disorders, and it presents limitations in terms of studying the complex interactions of multiple genes involved in oligogenic and multifactorial disorders. These complex interactions play important roles in most neurological diseases, such as non-syndromic neurodevelopmental disorders, migraine, cerebrovascular diseases. Thus, incorporating emerging technologies into molecular investigation holds the promise of enhancing the diagnostic capabilities.

**Table 2:** Summary of the limitations of whole-exome sequencing and proposed solutions

Limitation of WES	Explanation	Alternative approach
Limited coverage of non-coding regions	WES focuses only on exons, missing regulatory elements, introns, and intergenic regions	WGS to study non-coding regions or even targeted sequencing of regulatory elements
Challenges in identifying CNVs and other SVs	CNVs (deletions, and duplications), and other SVs may not be reliably detected because WES does not provide uniform coverage across regions	Use dedicated CNV detection tools alongside WES, implement WGS for better resolution of CNVs or long-read sequencing platforms to better identify structural variants
Difficulty with repetitive regions	Highly-repetitive regions (e.g., GC-rich regions) may not be adequately sequenced or accurately mapped	Employ long-read sequencing or integrate WES with targeted expansion studies
Incomplete coverage of all exons	Some exons are poorly captured due to probe design inefficiencies, leading to gaps in coverage	Optimize probe design and library preparation or use WGS
Inability to identify epigenetic modifications	Epigenetic factors, such as DNA methylation or histone modifications, are not studied by WES	Perform complementary epigenomic studies
Cost and computational requirements	Although cheaper than WGS, WES remains expensive for large-scale projects, and its data analysis requires significant computational resources	Employ targeted panel sequencing for cost-effective analysis of known genes or optimize bioinformatics tools
Interpretation of variants of VUS	WES often identifies numerous VUS, making clinical interpretation challenging due to limited functional information	Combine with functional assays, use updated standards for variant classification, and expand variant databases
Low resolution for mosaicism detection	WES has reduced sensitivity to detect low-level mosaicism in a sample.	Use ultra-deep sequencing or digital PCR to detect low-level mosaic variants
Limited access in public healthcare systems	In low- and middle-income countries, the lack of infrastructure and trained personnel often limits access to WES technology	Increase rare disease awareness and advocacy, expand diagnostic programs, and encourage public-private partnerships
High cost and lack of reimbursement	Many healthcare systems do not cover WES under insurance, and the high upfront cost remains a barrier to its adoption	Advocate for policy reforms, and expand coverage

Abbreviations: GC, guanine-cytosine; CNV, copy number variation; PCR, polymerase chain reaction; SV, structural variation; VUS, variants of unknown significance; WES, whole-exome sequencing; WGS, whole-genome sequencing.

The diagnostic rates using WES for the investigation of rare diseases are of approximately 38%, with notably higher rates observed among infants and children compared to adults. 19 Additionally, individuals with neurological disorders tend to present higher diagnostic rates. One effective strategy to enhance the diagnostic yield of WES is the simultaneous study of trios, consisting of the proband and both biological parents. This approach, known as trio sequencing, can improve the diagnostic yield by 10% to 15%. 81 The increased yield is primarily because trio sequencing facilitates the identification of de novo variants that neither parent possesses, which is an important molecular mechanism for several autosomal dominant disorders. Furthermore, for autosomal recessive disorders, it enables the confirmation of whether two variants within the same gene are biallelic, meaning they are located on both alleles of a gene in a compound heterozygous state.

Given that most patients do not receive a definitive diagnosis through initial WES, periodically reanalyzing the MPS data emerges as another strategy to increase diagnostic yields. Clinical studies 81 82 have demonstrated that periodic reevaluation can resolve an additional 10 to 15% of the cases per new analysis. This improvement is largely attributed to the discovery of novel gene-disease associations and updates on the pathogenicity of variants.

Genome sequencing, which analyzes all exons and approximately 90% of the genome, presents a potential to identify a wide array of genetic alterations that are often missed by ES. Genome sequencing has a better ability to detect smaller disease-causing CNVs and SVs, repeat expansions, and variants in nonexonic regulatory and splicing sites. 79 Additionally, genome sequencing improves variant calling in homologous sequences, which are regions of the genome with high similarity to each other, where WES might struggle more due to sequence alignment challenges. Studies 83 have demonstrated that genome sequencing can uncover diagnoses missed by WES in up to 14% of the cases, including disease-causing variants located deep within introns, CNVs, and SVs.

Experimental assessments employing specialized bioinformatics protocols tailored for genome sequencing and focusing on short tandem repeat (STR) expansions have been conducted and achieved remarkable accuracy in terms of identifying neurological disorders caused by STR expansions in several patients. 80 These include STRs located in noncoding regions, such as the 5′ untranslated region (that is, *C9orf72* , *FMR1* , and *PPP2R2B* ), 3′ untranslated region (that is, *DMPK* ), and intron 1 (that is, *FXN* ).

Another transformative technology in genomics has gained attention for its potential to increase our ability to read and understand genetic information: the long-read sequencing. This approach shows promising potential for more accurate detection of SVs and the study of genomic regions previously regarded as unreachable, such as complex and repetitive genomic regions, non-coding and regulatory elements, and the potential to study transcript isoforms and even epigenetic variations. 84

An innovative approach in molecular diagnosis, known as *multiomics* , involves integrating data from multiple omics disciplines, including genomics, transcriptomics, proteomics, metabolomics, and epigenomics. Combining big data from these various omics can provide crucial insights into biological markers, pathophysiology, and a more coherent understanding of the genotype-phenotype relationships in complex, unknown diseases. 85 However, integrating these large datasets into the clinical diagnostic scenario remains challenging and requires advanced bioinformatics protocols and expertise in data analysis.

An important public initiative has established a national pathway to embrace the next leap of genomics in Brazil. Funded by the Brazilian Health Ministry, the Brazilian Genomes Program commenced in 2020. Its primary objective is to create a comprehensive database of genomic data from 10 thousand Brazilians. The initiative includes various branches focused on different areas: rare diseases (the Rare Genomes Project), population genomics, and other clinical applications of genomics. The creation of representative databases of Brazilian genetic variants holds significant potential to improve the process of variant classification.

In conclusion, there have been numerous reports and valuable cohorts documenting the use of WES for the diagnosis of genetic disorders with neurological manifestations in Brazil, often emphasizing detailed phenotypic analyses and clinical presentations. The primary groups of diseases studied include neurodevelopmental disorders, hereditary spastic paraplegia, movement disorders, ataxia, dementia, myopathies, neuropathies, neuronopathies, and inherited metabolic diseases, among others. This molecular approach underscores the increasing importance of molecular diagnosis in neurology and highlights the growing interconnection between genetics and neurology. While WES has several limitations and may eventually be supplanted by more powerful diagnostic tools with higher diagnostic yields, the field of neurogenetics is set to become increasingly important in the future.
